# Contagion of Typhoid Fever

**Published:** 1852-07

**Authors:** 


*Contagion of Typhoid Fever.*—Dr. Piedvache, in the *Memoirs of the French Academy*, gives the following facts going to prove the contagious nature of this fever:

"1st. *Typhoid Fever, after attacking an individual, attacks other members of the same family in regular succession.* —From among M. Piedvache's 452 cases, in 49 there were two patients in the same house, in 30 three, in 14 four, in 13 five, in 2 six, in 1 seven, in 2 eight, and in 1 ten,—leaving but 92 (1-5), in which only one case was observed in the same house. The houses attacked nowise differed from those that escaped; and that the attacks of so many individuals in the same abodes did not result from mere local causes, is seen from the fact that such attacks were not simultaneous, three or four weeks always intervening between the first patient and his successors. The houses in which but one case occurred, were better than the others in regard to *ventilation*, as will be afterwards noticed.

2d. *An individual attacked with the fever, and transported to a family inhabiting a locality where it did not prevail, communicates it to that family.*—This is the fundamental proof relied upon by all those who have advocated the contagious nature of the disease, and especially by M. Gendrin (Journ. des Connaiss. Medic.," vols. i., and ii.), one of the ablest of these. This class of cases resembles the other in attacking several members of the same family; but the possibility of referring the propagation to any cause proper to the house which the patients inhabited, is quite cut away. The development of the disease too constantly followed the arrival of the patient to be regarded in the light of a coincidence; the family enjoying good health prior to such arrival, and giving no signs of the disease until three or four weeks after it.

3d. *An individual attacked by typhoid transmits it to the persons who are in immediate attendance upon him, while the rest of the family does not suffer.*—Several examples of this are cited, in which the disease occurred in localities where it was not prevalent, and among the more comfortably circumstanced portions of society. In the other category, in which the disease was transmitted to *several* members of the same family, the lower orders were alone those so affected.

4th. *The attendant to whom the fever has been communicated transmits it to others.*—Among the 452 cases of typhoid fever which form the ground-work of this essay 411 either transmitted the disease, or were themselves the product of transmission—that is, 9-10 of the entire number; while those cases in which no such transmission occurred, only confirm the author's views as to the mode in which such transmission was effected in the others.

Opposed to these statements, we have the *negative* ones advanced by some of the ablest physicians attached to the Parisian hospitals; but these cannot destroy the value of *positive* facts, and only show that there are circumstances under which the phenomenon of contagion is not exhibited. Even among M. Piedvache's own cases, while of the entire 452 transmission was proved in 411, it was so in 393 of 419 country cases, and only in 15 out of 33 which occurred in the town of Dinan, and some of these 15 lived in the environs; so that in the strictness they should be considered as rural cases also. Seeing the great difference as to the manifestation of contagion which occurs in town and country, the comparative condition of the patients in each should be inquired into. As peculiarities attaching to the rustic patients, M. Piedvache mentions the number of their visitors, and the great amount of personal attendance they receive from their neighbors; the bad construction and over-crowded state of their sleeping-rooms preventing effectual ventilation; and the utter neglect of all cleanliness,—though this last is of very secondary influence as compared with defective ventilation. It is obvious that in hospitals, and in houses of the gentry, where transmission of the disease is so rare, these conditions do not prevail. To the accidental obtaining a better ventilation, by sleeping in another room, the members of families among the poor have entirely owed their exemption when this has occurred; and to the more defective ventilation that is then procured, the author attributes the fact of more cases occurring in winter than in summer. He has never seen an example of contagion from a mere visit to a patient [the sojourn of a night in the patient's chamber seeming essential]; and priests and medical men, who are brought into very close *temporary* contact with the patient, very rarely acquire the disease.

An epidemic of typhoid does not spread from a centre, over a more or less considernble extent of country, like those of variola, scarlatina, &c. Only portions of a commune, a hamlet, or even a single house, may suffer; and once arrived in a locality, the length of its sojourn there is always remarkable. In the majority of the author's cases, the disease had reached the fourth week becoming communicated. It was so often later still, but very rarely so soon as the third week.

As several persons placed in favorable circumstances for acquiring the disease escaped doing so, some of the causes of their immunity may be adverted to. The first of these is the protection derived from a *prior attack.* M. Piedvache has never known an instance of its occurring a second time, and a patient surrounded by persons who have already had the disease does not propagate it. *Aged persons*, again, contract the disease with difficulty, though when exposed to circumstances favorable to contagion, they do not always escape. Among 138 cases, Louis found none above 39. Chomel supposed no case had been met with above 52; but since then Rayer has cited one of 56, and Prus one of 78. During the two epidemics witnessed by the author, almost all the aged persons living in houses where the disease prevailed escaped it. Still, 9 between 50 and 60, and 5 between 60 and 70, were attacked; and MM. Gendrin and Jacquet report similar examples. It was once believed that *young children* were very rarely attacked; but the researches of Rilliet and Barthez and Taupin show how erroneously. The attacks of children, in fact, often pass unperceived, and this may form some explanation of unlooked-for immunities in after life. Lastly, there are individuals whose constitution or idiosyncrasy resists the effect of contagion, under whatever circumstances they may be placed.—*Ibid.*

